# Controlateral epidural hematoma after VP shunt: A case report

**DOI:** 10.1016/j.amsu.2021.102663

**Published:** 2021-08-08

**Authors:** Yassine Tahrir, Said Hilmani, Abdelhakim Lakhdar

**Affiliations:** Neurosurgery Department, University Hospital Center IBN ROCHD, Casablanca, Morocco

**Keywords:** Epidural hematoma, Ventriculoperitoneal shunt, Brain, Surgery, Case report

## Abstract

Cerebrospinal fluid over-drainage is a common complication of ventriculoperitoneal devices (Ventriculoperitoneal shunt). In terms of hemorrhage, subdural hematomas are usually more frequent lesions than epidural hematomas, which, more rarely, may also be seen after ventricular shunt procedures and may lead to rapid neurological decline and even death unless a surgical procedure can be promptly performed. In our study we report the case of a 5 years old boy with history of congenital obstructive hydrocephalus treated with a ventriculoperitoneal shunt insertion when he was 8 months old. The patient was admitted with sudden deterioration of level of consciousness secondary to tri-ventricular hydrocephalus. He underwent a shunt revision. Two weeks later, he developed a loss of consciousness with a large left extradural hematoma contralateral to the side of ventriculoperitoneal shunt. He underwent an evacuation of the hematoma with a good postoperative outcome. Epidural hematoma, especially controlateral to Ventriculoperitoneal shunt, is extremely rare. The pathophysiology and the possible use of a programmable valve to prevent these lesions are briefly discussed.

## Highlights

• A 5 years old boy treated for congenital hydrocephalus since he was 8 months old with a fixed pressure Ventriculoperitoneal shunt on the right side. He was brought to our emergency for intracranial hypertension syndrome with disorders of consciousness. Patient's consciousness level was gradually improved after shunt revision. After 2 weeks, he was admitted with sudden deterioration of consciousness.
• The cerebral CT scan showed a massive left-sided epidural hematoma and was located in the right parietal and occipital aera, causing an important mass effect.
• The prognosis is good if the diagnosis is made quickly. The advent of programmable shunts (PS) theoretically enables a better way to avoid the over-shunting and its consequences.

## 1 Introduction

Ventriculoperitoneal shunt (VPS) is the most common technique used to long-therm management of hydrocephalus. However, this procedure is also beset with a considerable number of complications, which may be related to shunt obstruction, infection and excessive drainage of cerebrospinal fluid (CSF) [1], such as slit ventricle syndrome, formation of intracranial hematomas (subdural, epidural and intraventricular hematomas) [2]. Intracranial hemorrhages related to ventriculoperitoneal shunt surgery are important and possibly life-threatening complications. The main cause of these complications is over-drainage. Subdural bleeding after cerebrospinal fluid excessive drainage is the most common presentation [3]. However, the formation of epidural hematomas acutely after ventricular over-drainage is very rare and may be more life-threatening than subdural hematomas, with a reported fatality rate of 44.2% [4] and so, consequently should be promptly recognized and adequately approached.

## 2 Case report

A 5 years old boy treated for congenital hydrocephalus since he was 8 months old with a fixed pressure VPS on the right side. He was brought by his mother to our emergency for intracranial hypertension syndrome with disorders of consciousness. The CT scan showed active triventricular hydrocephalus.

Patient's consciousness level was gradually improved after shunt revision and was discharged 3 days later. After 2 weeks, he was admitted with sudden deterioration of consciousness. At admission, his GCS scores were 13/15 E3V4M5.

**Fig. 1:**
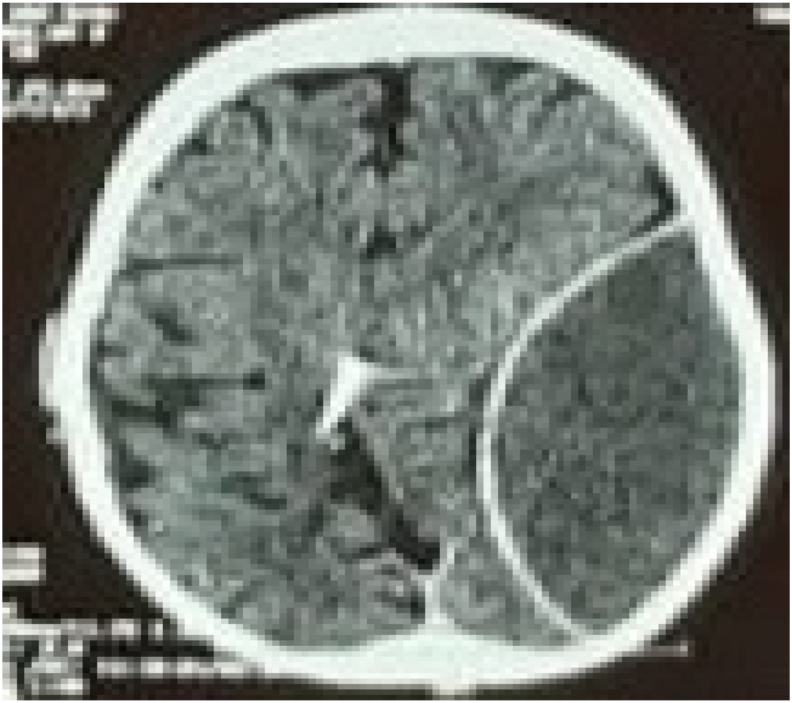
The CT scan of brain showing left extradural hematoma with mass effect. The ventricular catheter is seen within the right occipital horn.

Neurological examination revealed muscle power was 3/5 on right side, 5/5 on left side without sensitive trouble. The cerebral CT scan showed a massive left-sided epidural hematoma and was located in the right parietal and occipital aera, causing an important mass effect (Fig. 1).

**Fig. 2:**
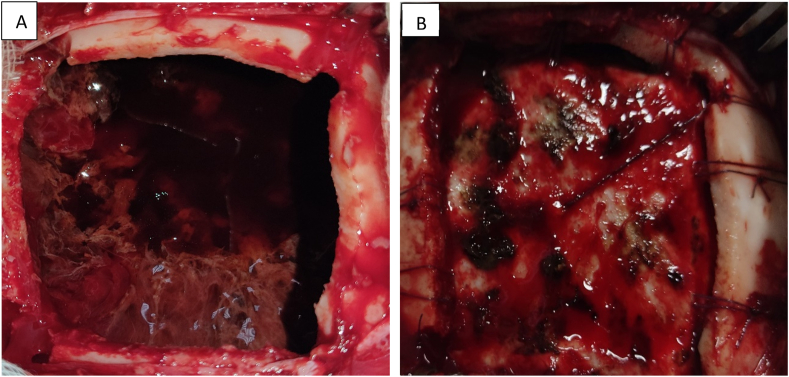
A/Intra operative images showing the epidural hematoma. B/ Tacking stitches to prevent the reaccumulation of blood within the epidural space.

The intervention was performed by our chief resident under general anesthesia a wide frontoparietal craniotomy was performed we proceeded to the evacuation of the epidural hematoma and tacked the dura up to the skull to prevent recurrence without shunt management (Fig. 2).

The follow up was good, he regains consciousness without any complication. At control CT scan we showed the absence of the epidural hematoma with normal aspect of the ventricles. The patient was discharged after 10 days.

the patient was seen after 1 month then 6 months and 1 year and the clinical examination was well without any anomaly confirmed by radiological controls.

This case has been reported in line with the 2020 SCARE guidelines {5}.

## 3 Discussion

The use of ventricular shunts to treat hydrocephalus is one of the most common neurosurgical procedures worldwide. The advent of such devices, despite many inconveniences and complications attributed to the use of a prosthesis, enables a fast and efficient treatment of hydrocephalus. Complications such as obstruction of the device and infection (up to 60%) are more frequent than bleeding [ 3,6].

Epidural hematomas are an unusual complication of ventriculoperitoneal shunt in the treatment of chronic hydrocephalus and are much less common than post shunting subdural hematomas [[5], [6], [7]]. The first case of epidural hematoma after ventricular drainage was made in 1941 [[6], [7], [8]]. Ever since, sporadic cases of epidural hematoma after ventricular overshunting has been reported [2,8], some of them as chronic/calcified lesions [9,10].

The mechanism of epidural hematoma formation is related to the rapid reduction in intracranial pressure as a result of overdrainage of the CSF, resulting in separation of the dura mater from the skull and tearing of small dural vessels [6,7,11,12]. According to Kalia [13], in some patients the skull-to-dura mater adhesion may be less strong than the dura mater-to-arachnoid adhesion, so that an epidural hematoma forms instead of the more common subdural hematoma. A possible hypothesis to explain the formation of contralateral epidural hematomas by overshunting would be the occurrence of traction forces over the middle meningeal artery and its branches (weakly adhered to the skull inner table) that follow the collapse of the brain tissue.

The most common location of epidural hematoma is on fronto-parietal lobes. This may be due to lose fixation of the dura to the cranial vault at this region [13]. Epidural hematoma tends to develop within the first few hours after the operation [8]. Besides causing neurological signs epidural hematoma can be life-threatening if not promptly suspected and treated.

The occurrence of epidural hematomas acutely (few days) after shunting, as in the case reported herein, is a rare condition and, in the authors opinion, demands immediate evacuation of the lesion as well as the prompt replace or, in the case of flow-controlled shunts, the reprogramming of the system pressure to higher levels.

The largest cases series of epidural hematoma after ventricular decompression, from Odake and Matsumoto [11], includes not only ventriculoperitoneal shunt but also other procedures for draining cerebrospinal fluid: ventricular puncture, ventriculography, ventriculoatrial shunts and so forth. According to them, epidural hematomas are more common in individuals of middle age (10–40 years). On the other hand, Kalia et al. [17] have reported the occurrence of multiple epidural hematomas after ventriculostomy in a child.

There are two distinct approaches for treatment of epidural hematomas in these cases; conservative and surgical, the choice for or against of which depend on clinical setting, age, time of bleeding, size on CT-scan, extent and thickness of the hematoma and the presence of midline shifting on CT. Any rapid accumulation in children and younger adults must be treated surgically.

Dominic Power, farouk ali-khan and Martin Drage report a historical case of a contralateral epidural hematoma after a ventricular shunt. They supposed that it was sudden decompression of the dilated ventricles that stripped the dura from the bone of the skull vault and caused the acute epidural hemorrhage [14]. The development of a fluid level within a pre-existing extradural hematoma has been reported after insertion of a VP shunt for hydrocephalus [18].

The particularity of our observation is that the epidural hematoma arose contralateral to the shunt without recent head trauma. We suppose that the mechanism appears to be caused by a combination of some factors: a likely over-functioning of the device was responsible for the hyper drainage with a sudden decompression of the dilated ventricles, stripping the dura from the bone and leading to the contralateral acute epidural hematoma. He only benefited from the evacuation of the hematoma which was compressive. As for the valve, we did not change it.

The advent of programmable shunts (PS) theoretically enables a better way to avoid the over-shunting and its consequences, once the width of the ventricles and the cerebrospinal fluid hydrodynamics can be strictly controlled by adaptation of the draining pressure. In fact, Carmel et al. [15] and Decq et al. [16] concluded that flow-controlled systems are useful to avoid mechanical failures and its consequences (mainly over-drainage) and to treat subdural collections when it occurs.

## 4 Conclusion

Controlateral epidural hematoma as a complication of ventriculoperitoneal shunt is a rare but rapidly evolving and often catastrophic entity. Maybe third ventriculocisternostomy or the use of programmable shunts and a longer hospital stay could change the evolution and outcome of these cases.

## Financial disclosure

The authors declared that this study has received no financial support.

## Ethical approval

Written informed consent was obtained from the patient for publication of this case report and accompanying images. A copy of the written consent is available for review by the Editor-in-Chief of this journal on request.

## Research registration unique identifying number (UIN)

None.

## Provenance and peer review

Provenance and peer review Not commissioned, externally peer-reviewed.

## Author contribution

Yassine TAHRIR: writing the paper and Corresponding author. Said HILMANI: Correcting the paper. Abdelhakim LAKHDAR: Correcting the paper.

## Guarantor

TAHRIR YASSINE.

## Declaration of competing interest

The authors of this article have no conflict or competing interests. All of the authors approved the final version of the manuscript.

## References

[1] Choux M., Genitori L., Lang D., Lena G. (1992). Shunt implantation: reducing the incidence of shunt infection. J. Neurosurg..

[2] Faulhauer K., Schmitz P. (1978). Overdrainage phenomena in shunt treated hydrocephalus. Acta Neurochir..

[3] Murata T., Shigeta H., Horiuchi T., Sakai K., Hongo K. (2010). Globular subdural hematoma in a shunt-treated infant: case report. J. Neurosurg. Pediatr..

[4] Odake G., Matsumoto S. (1981). Supratentorial epidural hematoma as a complication of internal decompression–one personal and 42 reported cases. Neurol. Med.-Chir..

[5] Agha R.A., Franchi T., Sohrabi C., Mathew G., for the SCARE Group (2020). The SCARE 2020 guideline: updating consensus surgical CAse REport (SCARE) guidelines. Int. J. Surg..

[6] Louzada P.R., Requejo P.R., Barroso M.V., Vaitsman R.P., Machado A.L., Paiva M.S., Salame J.M. (2012). Bilateral extradural haematoma after acute ventricular over-drainage. Brain Inj..

[7] Aguiar P., Shu E.S., Freitas A. (2000). Causes and treatment of intracranial haemorrhage complicating shunting for paediatric hydrocephalus. Child's Nerv. Syst..

[8] Driesen W., Elies W. (1974). Epidural and subdural haematomas as a complication of internal drainage of cerebrospinal fluid in hydrocephalus. Acta Neurochir..

[9] Gulliksen G., Haase J. (1977). Epidural hematoma following a shunt revision. Acta Neurochir..

[10] Murata T., Shigeta H., Horiuchi T., Sakai K., Hongo K. (2010). Globular subdural hematoma in a shunt-treated infant: case report. J. Neurosurg. Pediatr..

[11] Odake G., Matsumoto S. (1981). Supratentorial epidural hematoma as a complication of internal decompression–one personal and 42 reported cases. Neurol. Med.-Chir..

[12] Moussa A.H., Sharma S.K. (1978). Subdural haematoma and the malfunctioning shunt. J. Neurol. Neurosurg. Psychiatry.

[13] Higazi I. (1963). Epidural hematoma as complication of ventricular drainage: report of a case and review of literature. J. Neurosurg..

[14] Power D., Ali-Khan F., Drage M. (1999 Jul). Contralateral extradural haematoma after insertion of a programmable-valve ventriculoperitoneal shunt. J. R. Soc. Med..

[15] Carmel P.W., Albright A.L., Adelson P.D., Canady A., Black P., Boydston W., Kneirim D., Kaufman B., Walker M., Luciano M. (1999). Incidence and management of subdural hematoma/hygroma with variable- and fixed-pressure differential valves: a randomized, controlled study of programmable compared with conventional valves. Neurosurg. Focus.

[16] Decq P., Barat J.L., Duplessis E., Leguerinel C., Gendrault P., Keravel Y. (1995). Shunt failure in adult hydrocephalus: flowcontrolled shunt versus differential pressure shunts–a cooperative study in 289 patients. Surg. Neurol..

[17] Kalia K.K., Swift D.M., Pang D. (1993). Multiple epidural hematomas following ventriculoperitoneal shunt. Pediatr. Neurosurg..

[18] Iplikçioglu A.C., Bayar M.A., Kokes F., Yildiz B., Gokçek C., Buharali Z. (1994). A fluid level in an acute extradural haematoma. Neuroradiology.

